# Theory of visual attention (TVA) applied to rats performing the 5-choice serial reaction time task: differential effects of dopaminergic and noradrenergic manipulations

**DOI:** 10.1007/s00213-022-06269-4

**Published:** 2022-11-25

**Authors:** Mona El-Sayed Hervig, Chiara Toschi, Anders Petersen, Signe Vangkilde, Ulrik Gether, Trevor W. Robbins

**Affiliations:** 1 Department of Psychology and Behavioural and Clinical Neuroscience Institute, University of Cambridge, Cambridge, UK; 2 Department of Neuroscience, University of Copenhagen, Copenhagen, Denmark; 3 Department of Psychology, University of Copenhagen, Copenhagen, Denmark

**Keywords:** Attention, Amphetamine, Methylphenidate, Atomoxetine, Atipamezole, Phenylephrine, ADHD, TVA, Visual processing

## Abstract

**Rationale:**

Attention is compromised in many psychiatric disorders, including attention-deficit/hyperactivity disorder (ADHD). While dopamine and noradrenaline systems have been implicated in ADHD, their exact role in attentional processing is yet unknown.

**Objectives:**

We applied the theory of visual attention (TVA) model, adapted from human research, to the rat 5-choice serial reaction time task (5CSRTT) to investigate catecholaminergic modulation of visual attentional processing in healthy subjects of high- and low-attention phenotypes.

**Methods:**

Rats trained on the standard 5CSRTT and tested with variable stimulus durations were treated systemically with noradrenergic and/or dopaminergic agents (atomoxetine, methylphenidate, amphetamine, phenylephrine and atipamezole). TVA modelling was applied to estimate visual processing speed for correct and incorrect visual perceptual categorisations, independent of motor reaction times, as measures of attentional capacity.

**Results:**

Atomoxetine and phenylephrine decreased response frequencies, including premature responses, increased omissions and slowed responding. In contrast, methylphenidate, amphetamine and atipamezole sped up responding and increased premature responses. Visual processing speed was also affected differentially. Atomoxetine and phenylephrine slowed, whereas methylphenidate and atipamezole sped up, visual processing, both for correct and incorrect categorisations. Amphetamine selectively improved visual processing for correct, though not incorrect, responses in high-attention rats only, possibly reflecting improved attention.

**Conclusions:**

These data indicate that the application of TVA to the 5CSRTT provides an enhanced sensitivity to capturing attentional effects. Unexpectedly, we found overall slowing effects, including impaired visual processing, following drugs either increasing extracellular noradrenaline (atomoxetine) or activating the α1-adrenoceptor (phenylephrine), while also ameliorating premature responses (impulsivity). In contrast, amphetamine had potential pro-attentional effects by enhancing visual processing, probably due to central dopamine upregulation.

**Supplementary Information:**

The online version contains supplementary material available at 10.1007/s00213-022-06269-4.

## Introduction

Attentional capacity is essential in the interaction with our surroundings, where we need to swiftly select the most relevant sensory information for conscious perception, while discounting irrelevant stimuli (Cohen 2014). Compromised attention is a hallmark of a range of psychiatric disorders, including attention-deficit/hyperactivity disorder (ADHD) (Arnsten 2006), but the neurobiological foundation of attention and how current pharmacological treatments, targeting dopaminergic and noradrenergic neural systems, act on this psychological construct still remains to be understood.

In humans, the theory of visual attention (TVA) model (Bundesen 1990; Bundesen et al. 2005) has been used to study attentional capacity in pharmacological studies and different clinical conditions, including ADHD (Habekost 2015). In this mathematical model, a set of simple equations describes visual attention mechanisms, including processing speed of visual perceptual categorisations, which is a measure of attentional processing independent of motor reaction time (Bundesen 1990; Habekost and Starrfelt 2009; Bundesen et al. 2015). To enable translational studies on attention, the TVA model was recently adapted to model effects of the anti-cholinergic drug scopolamine in mice in a 5-choice serial reaction time task (5CSRTT) (Fitzpatrick et al. 2017)—a well-established task used to assess attention and impulsivity in rodents (Robbins 2002). We now adapt the TVA model to the rat 5CSRTT to study dopaminergic and noradrenergic modulation of attentional processing capacity in rats. We increased attentional load by making target stimuli temporally, as well as spatially, unpredictable using a variable stimulus duration (vSD) regimen. This vSD-5CSRTT combined with the TVA model (TVA-5CSRTT) is equivalent to previous human TVA paradigms with single-letter exposures (Bundesen and Harms 1999) and provides an exact and reliable prediction of attentional capacity with high translational value (Habekost and Starrfelt 2009; Habekost et al. 2014) that has never previously been used to study catecholaminergic modulation of visual attentional processing in rats.

Methylphenidate (MPH), amphetamine (AMPH) and atomoxetine (ATO) are commonly used to treat ADHD (Bidwell et al. 2011). However, the specific, and potentially dissociable, effects of these compounds on attention remain unclear. Furthermore, inconsistencies in their effects exist between preclinical and clinical studies, supporting the need for improved translational investigation. In healthy humans, the stimulant drugs, MPH and AMPH, apparently improve ‘processing speed accuracy’ (Marraccini et al. 2016) and TVA modelling has shown that MPH improves visual processing speed in participants with low baseline attentional performance (Finke et al. 2010). Despite clinical studies indicating cognitive enhancing effects of stimulants, studies using the standard 5CSRTT in intact rodents generally fail to find consistent pro-attentional effects on the accuracy variable after clinically relevant (low to moderate) doses of MPH (Navarra et al. 2008; Milstein et al. 2010) and AMPH (Cole and Robbins 1987; Harrison et al. 1999; Van Gaalen et al. 2006; Loos et al. 2010; Balachandran et al. 2018). However, some have reported stimulants to improve attention in low-attention (Robinson 2012; Caballero-Puntiverio et al. 2017) and high-impulsive (Caprioli et al. 2015) rodents as well as with a variable ITI challenge (Toschi et al. 2021), while impairing attention in rats with profound forebrain NA depletion (Cole and Robbins 1987).

The NA reuptake inhibitor, ATO, consistently improves attention in clinical ADHD studies (Wilens et al. 2006; Faraone and Glatt 2010; Hazell et al. 2011), but only a few studies have assessed attentional effects of acute ATO in healthy humans. These have shown that ATO improves rapid visual information processing (Crockett et al. 2010), but has no effect on attentional performance in the stop-signal reaction time task (Chamberlain et al. 2007) or in a recent human CombiTVA study (Lansner 2022). Likewise, results in rodents have been diverse depending on task and attentional phenotype. While 5CSRTT studies with ATO have shown no effects on accuracy in rodents (Robinson et al. 2008; Fernando et al. 2012; Sun et al. 2012; Pillidge et al. 2014), including an ADHD-like mouse model (Pillidge et al. 2014), some studies have shown improved accuracy in rodents with low attentional baselines (Robinson 2012) or if challenged with vSD (Callahan et al. 2019).

Previous work in rats and monkeys suggest prefrontal cortical noradrenergic α2-adrenoceptor involvement in beneficial effects of MPH and ATO in attention (Arnsten and Dudley 2005; Gamo et al. 2010). Guanifacine, an α2-adrenoceptor agonist, has been approved as an ADHD medication (Bidwell et al. 2011), and blocking the α2-adrenoceptor has produced phenotypes similar to ADHD in monkeys (Arnsten and Li 2005). However, studies are inconsistent in regard to its beneficial effects on attention, as some studies have shown impaired attentional performance after α2-adrenoceptor agonist administration in humans (Smith and Nutt 1996; Coull et al. 2004) and rats (Sirviö et al. 1994; Ruotsalainen et al. 1997; Brown et al. 2012; Fernando et al. 2012), while blocking the α2-adrenoceptor has been reported to improve attention in humans (Mervaala et al. 1993) and rats performing different attention tasks (Sirvio et al. 1993; Koskinen et al. 2003; Lapiz and Morilak 2006; Brown et al. 2012; Bari and Robbins 2013). Previous studies have also indicated a role for the noradrenergic α1-adrenoceptor in attention, for instance by increasing vigilance (Sirviö and MacDonald 1999). Only a few 5CSRTT studies have investigated the role of α1-adrenoceptors in attention, with some indicating α1-adrenoceptor activation to improve attention (Puumala and Sirviö 1997), while others reported no attentional effects (Pattij et al. 2012). In the present study, we used atipamezole (ATI), an α2-adrenoceptor antagonist and phenylephrine (PHEN), an α1-adrenoceptor agonist, to further investigate the role of α2- and α1-adrenoceptors in visual attentional processing.

Despite increasing insights into the role of DA and NA in attention, it is still unknown how these functions translate into quantitative measures of visual attention. In the current study, the TVA model adapted from human studies in attention was applied to the well-established 5CSRTT paradigm in rats to assess the effects of pharmacological challenges of the DA and NE systems on visual attentional processing in healthy rats. We hypothesised that the quantitative parameters assessed in this paradigm reflect mental psychological processes that are differentially modified by pharmacological challenges targeting the DA and NA transmitter systems.

## Methods and materials

### Animals

Outbred male Lister Hooded rats (*N* = 24; Charles River, Margate, UK) weighing 280–300 g at the beginning of the experiments were used. Animals were allowed to acclimatise to the animal facility under a 12-h:12-h light cycle (lights off at 7 AM) for a minimum of 7 days before any procedures began. When rats reached a body weight of approximately 300 g, they were food restricted to maintain approximately 90% of their free-feeding weight trajectory (19 g of Purina rodent chow per animal and day; adjusted for reward pellet consumption during testing). Water was available ad libitum and food was given at the end of each day’s testing. This research has been regulated under the Animals (Scientific Procedures) Act 1986 Amendment Regulations 2012 (Project licence PA9FBFA9F held by Dr AL MIlton) following ethical review by the University of Cambridge Animal Welfare and Ethical Review Body.

### Drugs

Methylphenidate hydrochloride (1 mg/kg; Johnson Matthey, Edinburgh, UK), atomoxetine hydrochloride (1 mg/kg; Sigma-Aldrich, Dorset, UK), d-amphetamine humisulfate salt (0.2 mg/kg; Sigma-Aldrich, Dorset, UK), atipamezole hydrochloride (0.3 mg/kg; Abcam, Cambridge, UK) and phenylephrine hydrochloride (1 mg/kg; Sigma-Aldrich, Dorset, UK) were dissolved in 0.9% saline to 1 ml/kg fresh on the day of testing.

Doses were determined based on an extensive previous literature employing dose–response studies on the 5CSRTT and relevant behavioural tasks as well as further piloting. In general, we were interested in behaviourally relevant doses and attempted to choose as low doses as possible to avoid disruptive effects and to simulate likely clinical dosage. It is a limitation of the design that we were unable to obtain detailed dose–response data for all compounds; however, this constraint was pragmatically necessary to obtain data on the range of drugs investigated. The 1 mg/kg MPH dose was chosen based on previous dose–response studies using doses ranging around 0.3–3 mg/kg showing moderate doses around 1 mg/kg to improve attention under increased task demand (e.g. Koffarnus and Katz 2011; Berridge et al. 2006; Tomlinson et al. 2014; Navarra et al. 2017), while higher doses potentially would impair performance by inducing disruptive impulsivity (e.g. Milstein et al. 2010). Furthermore, we conducted a dose–response study on the effects of 1 and 3 mg/kg MPH on a vITI-5CSRTT paradigm showing that 1 mg/kg (as opposed to 3 mg/kg) MPH improved performance (more rewards earned) on the 5 s ITI (the ITI used in the present study), while not increasing premature responding to the same extend as 3 mg/kg MPH (Toschi et al. 2021). The 0.2 mg/kg AMPH dose was chosen based on a previous dose–response study showing improved attention in a signal detection task after an equivalent low-dose AMPH, as opposed to higher doses (1.25 mg/kg), which impaired attention (Turner and Burne 2016). We further tested the 0.2 mg/kg AMPH dose in our recent publication on vITI-5CSRTT showing improved attention selectively at short (3 s) ITIs (Toschi et al. 2021). The 1 mg/kg ATO dose was chosen based on dose–response studies using doses ranging around 0.3–3 mg/kg (e.g. Benn and Robinson 2017; Ding et al. 2018; Baarendse and Vanderschuren 2012; Callahan et al. 2019; Fernando et al. 2012; Robinson et al. 2008; Koffarnus and Katz 2011, Tomlinson et al. 2014). We chose the moderate dose of 1 mg/kg ATO, which consistently improves impulsivity (e.g. Toschi et al. 2021; Navarra et al. 2008; Higgins et al. 2020), with some potential to affect accuracy (e.g. Navarra et al. 2008; Tomlinson et al. 2014) without extensively disrupting performance which 3 mg/kg ATO would potentially do (e.g. increased omissions in Koffarnus and Katz 2011). For ATI, we based our dose on previous dose–response studies employing doses in the range of around 0.03–1 mg/kg ATI showing 0.3 mg/kg ATI to improve attention (Sirviö et al. 1993) and stop-signal reaction time (Bari and Robbins et al. 2013). The PHEN dose was based on a previous 5CSRTT paper with extensive data on dose–response (Pattij et al. 2012) and further piloting, where we first tested the 3 mg/kg as it indicated some improvement in Pattij et al., but as this was sedative in our pilot rats, we chose the 1 mg/kg dose, where the rats could perform the task.

### 5-choice serial reaction time task (5CSRTT)

#### Apparatus

Details of the behavioural apparatus have been provided previously (Bari et al. 2008). In brief, we used twelve five-choice operant chambers (Med Associates Inc., St. Albans, USA) each contained within a ventilated and sound-attenuated chamber. Each chamber comprised five evenly and distinctly spaced apertures containing an LED light set into a curved wall at the rear of the chamber. On the opposite wall of the chamber, a central food magazine was located, into which 45-mg reward pellets could be delivered (TestDiet 5UTL, Sandown Scientific, Middlesex, UK). Infrared beams located at the entrance of each aperture and the food magazine allowed the detection of nose pokes. The chambers were controlled by computers using WhiskerServer and FiveChoice client software (Cardinal and Aitken, 2010).

#### Pretraining: 5-choice serial reaction time task (5CSRTT)

All rats were trained in the 5CSRTT as described in detail previously (Bari et al. 2008). In short, animals were trained through progressing training stages (as described in Bari et al. 2008) to detect a brief visual cue appearing pseudorandomnly in one of five apertures of the rear wall of the operant chamber. Each trial was initiated by the rat nose poking into the food magazine and the visual cue is presented after an intertrial interval (ITI) of 5 s. A response was deemed ‘correct’ if the animal nose-poked into the hole with the visual stimulus. A nose-poke response occurring before the appearance of the visual cue was considered ‘premature’, while one occurring in any of the other apertures without the visual cue was considered ‘incorrect’. A failure to respond within 5 s (limited hold) of target presentation was recorded as an ‘omission’. Correct responses were rewarded with one food pellet, while incorrect, premature and omission responses were punished with a time-out (TO) period of 5 s, following which another trial could be initiated. Nose pokes in any of the apertures made after a correct or incorrect response, but prior to reward collection, were deemed ‘perseverative’ but were not signalled by punishment (i.e. TO). Each training and baseline session lasted maximum 100 trials or 30 min, whichever was reached first. In this cohort of rats, a stable baseline performance on the 5CSRTT was reached at training stage 11 (Bari et al. 2008), i.e. stimulus duration of 600 ms and an ITI of 5 s, where > 80% accuracy and < 20% omissions was reached.

#### Variable stimulus duration challenge (vSD-5CSRTT)

For testing drug effects on behaviour, the rats were challenged with a variable stimulus duration (vSD) schedule with fixed 5 s ITI schedule in a prolonged session of 60 min, or 200 trials, to allow for sufficient trials at each SD. In this schedule, the SDs (75, 150, 300, 600 and 1200 ms) were presented pseudorandomly in blocks of 50, offering both increased and decreased task difficulty within-session compared with the baseline conditions (Fig. S1). Both the variable nature of the schedule and the inclusion of short SD challenges the attention of the rats sufficiently to allow for the detection of potential attention-enhancing drug effects, and the vSD schedule also allows for TVA modelling to be applied. A pilot study was performed to select these SDs that increased the attentional load while still maintaining high motivation levels throughout the session.

#### Behavioural testing and drug administration

Drugs were administered sub-cutaneously 40 min prior to testing the animals on a vSD session of the 5CSRTT. The experiment consisted of two separate within-subject cross-over Latin-square designs, to control for training and cross-over effects. These two Latin-square designs, and each of the testing days, were separated by at least 3 days of washout and re-baseline sessions. In Latin-square 1: vehicle, AMPH (0.2 mg/kg), MPH (1 mg/kg) and ATO (1 mg/kg) were administered. In Latin-square 2: vehicle, ATI (0.3 mg/kg) and PHEN (1 mg/kg) were administered. Out of the initial 24 rats, 23 rats took part in the Latin-square 1 design, while 22 rats took part in the following Latin-square 2 design. This was due to two rats experiencing spontaneuous seizures: one rat being euthanised during pretraining, and another rat being euthanised between the two experiments.

### TVA modelling of 5-CSRTT data (TVA-5CSRTT)

Based on the framework of human TVA (Bundesen 1998; Bundesen and Harms 1999; Bundesen et al. 2005; Habekost 2015) and the recently developed TVA model for mouse 5CSRTT (Fitzpatrick et al. 2017), we developed a four-parameter TVA model adapted to the rat 5CSRTT. This makes it possible, for the first time, to estimate visual perceptual processing speed independent of motor reaction times from rat 5CSRTT data. TVA describes visual attention as a parallel processing race where different visual perceptual categorisations of a stimulus compete for entrance into visual short-term memory. For the rat 5CSRTT, we assume that one correct categorisation races against four incorrect categorisations. This is an extension of the TVA model for mouse 5CSRTT where only correct categorisations were modelled as only very few incorrect responses were observed for the mice. The rat TVA model thus focuses in greater detail on the perceptual processes compared with the mouse TVA model.

We assume that the rat makes a correct motor response if a correct visual perceptual categorisation finishes first and before time $\tau {,}$ where $\tau$ is the stimulus duration. In contrast, the rat makes an incorrect motor response if an incorrect visual perceptual categorisation finishes first and before time $\tau$. The sampling time for both correct and incorrect categorisations are assumed exponentially distributed with rate parameter ${\nu }_{\mathrm{c}}$ and ${\nu }_{\mathrm{i}}$, respectively, but delayed by a constant ${t}_{0}$, which is the time it takes the rat to orient toward the stimulus and initiate the race. If no correct or incorrect categorisation is made before time $\tau {,}$ we assume that with a certain probability (${p}_{\mathrm{g}}$) the rat guesses randomly among the five possible responses. An omission occurs if no categorisation is made before time ${\tau}$ and the rat does not choose to make a random response. In total, the TVA model for rat 5CSRTT has four free parameters: ${\nu }_{\mathrm{c}}$, ${\nu }_{\mathrm{i}}$, ${t}_{0}$ and ${p}_{\mathrm{g}}$. The probabilities of making a correct response, ${p}_{\mathrm{c}}$, an incorrect response, ${p}_{\mathrm{i}}$, or an omission, ${p}_{\mathrm{o}}$, are calculated as follows:

If $$\begin{aligned}p_{\mathrm c}=\int_0^{\tau-t_0}\nu_{\mathrm c}e^{-\nu_{\mathrm c}t}\cdot e^{-4\nu_{\mathrm i}t}dt+e^{-\nu_{\mathrm c}(\tau-t_0)}\cdot e^{-4\nu_{\mathrm i}\left(\tau-t_0\right)}\cdot p_{\mathrm g}\cdot\frac{1}{5}=\frac{\nu_{\mathrm c}}{\nu_{\mathrm c}+4\nu_{\mathrm i}}\left(1-e^{-\left(\nu_{\mathrm c}+4\nu_{\mathrm i}\right)\cdot\left(\tau-t_0\right)}\right)+e^{-\left(\nu_{\mathrm c}+4\nu_{\mathrm i}\right)\cdot\left(\tau-t_0\right)}\cdot p_{\mathrm g}\cdot\frac{1}{5}\\ p_{\mathrm i}=\int_0^{\tau-t_0}\nu_{\mathrm i}e^{-4\nu_{\mathrm i}t}\cdot e^{-\nu_{\mathrm c}t}dt+e^{-\nu_{\mathrm c}(\tau-t_0)}\cdot e^{-4\nu_{\mathrm i}\left(\tau-t_0\right)}\cdot p_{\mathrm g}\cdot\frac{1}{5}=\frac{\nu_{\mathrm i}}{\nu_{\mathrm c}+4\nu_{\mathrm i}}\left(1-e^{-\left(\nu_{\mathrm c}+4\nu_{\mathrm i}\right)\cdot\left(\tau-t_0\right)}\right)+e^{-\left(\nu_{\mathrm c}+4\nu_{\mathrm i}\right)\cdot\left(\tau-t_0\right)}\cdot p_{\mathrm g}\cdot\frac{1}{5}\\p_{\mathrm o}=e^{-\left(\nu_{\mathrm c}+4\nu_{\mathrm i}\right)\cdot\left(\tau-t_0\right)}\cdot\left(1-p_{\mathrm g}\right)\end{aligned}$$

If $$\tau \leq {t}_{0}$$, then

$$p_{\mathrm c}=p_{\mathrm g}\cdot\frac{1}{5} p_{\mathrm i}=p_{\mathrm g}\cdot\frac{1}{5} p_{\mathrm o}=\left(1-p_{\mathrm g}\right)$$

**Fig. 1:**
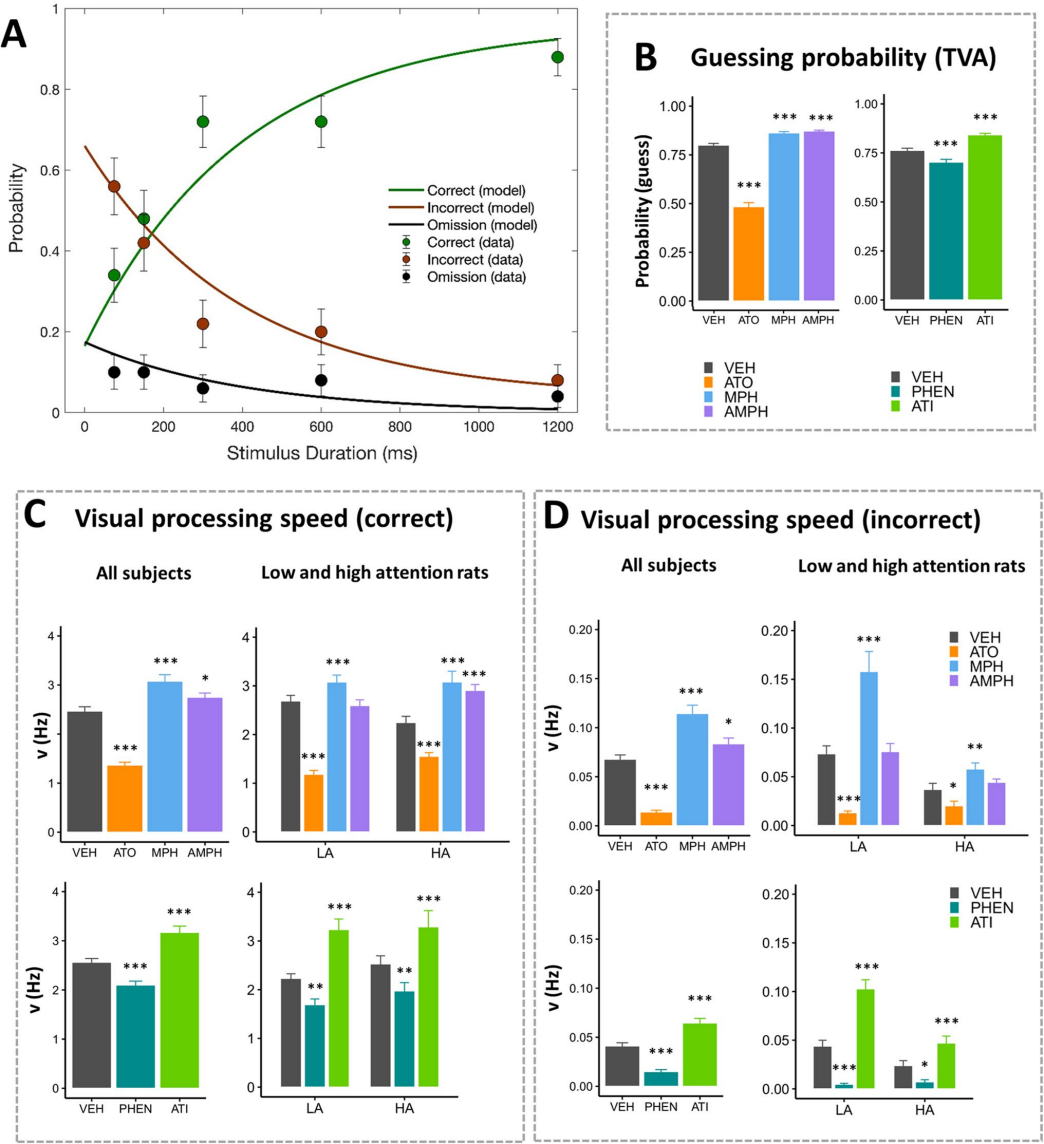
Effects of stimulant and non-stimulant drugs on modelled TVA-5CSRTT parameters. **A** The TVA model fitted to representative data from a rat performing a vSD challenge session. **B**–**D** presents results for Latin-square 1 (LS1, top panels) and Latin-square 2 (LS2, bottom panels) with TVA-modelled parameters willingness to guess (*p*_g_) (**B**), visual processing speed for correct responses (ν_c_) (**C**) and visual processing speed for incorrect responses (ν_i_) (**D**). LA, low-attention rats; HA, high-attention rats; ATO, atomoxetine; MPH, methylphenidate; AMPH, amphetamine; PHEN, phenylephrine; ATI, atipamezole. Results are represented as mean ± SEM; ***p < 0.001; **p < 0.01; *p < 0.05

TVA parameters were estimated by performing a maximum-likelihood fitting procedure using the Nelder-Mead simplex optimisation algorithm in Matlab 2017. Figure 1A shows the TVA model fitted to representative data from a rat performing a vSD-5CSRTT challenge session. We assessed the goodness-of-fit based on a pseudo-*R*^2^ (Nagelkerke 1991), which was calculated as

$${R}^{2}=\frac{1-{\mathrm{exp}({LL}_{0}-{LL}_{1})}^{2/n}}{{\mathrm{exp}({LL}_{0})}^{2/n}}$$

in which *LL*_0_ is the log-likelihood of the restricted model, *LL*_1_ is the log-likelihood of a less restricted model, and *n* is the number of trials. We set the *LL*_1_ as the log-likelihood of the TVA model and *LL*_0_ as the likelihood of a null model with only ${p}_{\mathrm{g}}$ as parameter. Thus, this pseudo-*R*^2^ reflects the proportion of variation explained by the TVA model relative to a null model with constant propabilities of making a correct response, an incorrect response and an omission across all stimulus durations. For Latin-square 1, the average ${R}^{2}$ s were 0.69, 0.69, 0.69 and 0.57 for vehicle, AMPH, MPH and ATO, respectively. For the Latin-square 2, the average ${R}^{2}$ s were 0.71, 0.72 and 0.66 for vehicle, ATI and PHEN, respectively. Altogether, this shows that the TVA model explains a large proportion of the variation in the data.

### Data analysis

Main 5CSRTT parameters of interest were correct responses, choice accuracy (% correct/(correct + incorrect)), omissions, premature responses, response latency to make a correct response after the onset of the target stimulus (ms) and latency to collect food from the magazine after a correct trial (ms). We also analysed incorrect responses, perseverative responses and response latency to make an incorrect response (as shown in supplementary Figs. S2-S3). TVA parametres of interest were visual processing speed for correct (${\nu }_{\mathrm{c}}$) and incorrect (${\nu }_{\mathrm{i}}$) responses and guessing probability (${p}_{\mathrm{g}}$). Based on mean accuracy score averaged across 13 separate days of baseline training, we classified low attention (LA) and high attention (HA) subgroups as the lower and upper 30th percentiles, respectively (*n* = 7 per subgroup).

Visualisation and statistical tests were performed with RStudio, version 1.2.1335 (RStudio, Inc.). Response frequencies (correct and incorrect responses, omissions and premature responses) were square-root transformed, latencies were log transformed and probabilities (accuracy and *p*_g_) were arcsin transformed to ensure normality, as confirmed with a quantile–quantile plot of residuals. Within each Latin-square design, differences in drug effects on the above parameters were analysed using linear mixed-effects model analysis with the lmer package in *R*. The model contained either one fixed factor (dose) or two fixed factors (drug and phenotype) and one random factor (subject; to account for individual differences between rats). When relevant, further analyses were performed by conducting separate multilevel models on ‘drug’ for each phenotype. These analyses were followed by post hoc Dunnet’s corrected pairwise comparisons with vehicle. We also ran models with two fixed factors being drug and SD, and three fixed factors being drug, phenotype and SD, but without finding significant interactions. Because drug effects were not dependent on SD, it is not included here. Linear correlations between TVA parameters and standard parameters were performed using Pearson’s coefficient r. Significance was set at α = 0.05.

## Results

No drugs affected accuracy, but ATO and PHEN decreased correct and incorrect responses, increased omissions and slowed responding. In contrast, ATI, MPH and AMPH did not affect correct or incorrect response frequencies, but speed up responding and increased premature responding. TVA-modelled visual processing speed was also affected differentially. While ATO and PHEN slowed, ATI, MPH and AMPH speed up, visual processing, both for correct and incorrect categorisations. AMPH selectively improved visual processing for correct, not incorrect, responses in high-attention rats only, reflecting improved attention. For readability, statistical details on pairwise comparisons are presented in Tables 2 and 3.

### Performance with variable stimulus duration (vSD) challenge

Behavioural performance in vehicle-treated rats during the vSD challenge was examined (Fig. S1) (see supplementary material for statistical details), but in short, performance improved with increasing SDs, i.e. increasing accuracy and correct responses as well as decreasing incorrect responses, omissions and response latencies, while leaving reward collection latency unaffected. Thus, attentional performance overall improved with increasing SDs and was dependent on SD, as expected (Fitzpatrick et al. 2017).

### Correlation between standard and TVA-modelled parameters in the 5CSRTT

To investigate how TVA-modelled parameters relate to standard 5CSRTT parameters, we analysed correlations in VEH groups averaged across Latin-square experiments (mean VEH; Tables 1 and S1) (for statistical details, see Table 1). The TVA-modelled ν_c_ parameter correlated positively with correct responses and negatively with omissions, while it did not significantly correlate with any other parameters such as, e.g. latencies, accuracy and premature responses. This indicated higher ν_c_ to be associated with enhanced correct responding and task engagement independent of errors and motor reaction times. TVA-modelled ν_i_ correlated negatively with omissions and positively with perseverative nosepokes. Thus, higher ν_i_ was associated with higher task engagement and increased (unrewarded) perseverative responding. *p*_g_ correlated positively with incorrect responses and negatively with accuracy and omissions. Thus, higher willingness to ‘guess’ was associated with more errors, lower accuracy and higher task engagement. Furthermore, ν_c_ and ν_i_ correlated positively, and *p*_g_ correlated positively with both ν_c_ and ν_i_, reflecting that higher ν_c_ is associated both with higher ν_i_ and willingness to guess (Table S1). Drug treatments affected these associations differentially, as described to some detail below and in supplementary material (Table S1).

**Table 1:** Pearson *R* correlations between TVA-modelled and standard 5CSRTT parameters in vehicle-treated rats

Mean VEH	ν_c_		ν_i_		*p*_g_	
	Pearson *R*	*p*-value	Pearson *R*	*p*-value	Pearson *R*	*p*-value
Accuracy	0.15	0.48	− 0.13	0.56	− 0.6	0.0022**
Correct responses	0.53	0.0088**	0.27	0.23	0.19	0.39
Omissions	− 0.66	5.7e-4***	− 0.55	0.0079**	− 0.97	2.2e-14***
Premature responses	0.038	0.86	0.13	0.56	0.18	0.42
Latency correct	− 0.37	0.081^#^	− 0.1	0.65	− 0.23	0.29
Latency collect	− 0.27	0.21	− 0.38	0.083^#^	− 0.39	0.065^#^

Visual processing speed (Hz) for correct (**ν**_**c**_) and incorrect (**ν**_**i**_) responses. *P*_g_, probability of guessing when no information is available. *p*, calculated probability (*p*-value)

### Effects of atomoxetine, methylphenidate and amphetamine on modelled TVA parameters

For ν_c_ (Fig. 1C and Table 2), we found a significant effect of drugs overall (*F*_3, 618_ = 226.09, *p* < 0.0001); ν_c_ was significantly decreased by ATO and increased by both MPH and AMPH. We found a significant drug × phenotype interaction (*F*_3, 372_ = 8.77, *p* < 0.0001), and significant main effect of drugs (*F*_3, 372_ = 139.77, *p* < 0.0001), but not of phenotype. Significant drug effects were present both in LA (*F*_3, 186_ = 130.42, *p* < 0.0001) and HA (*F*_3, 186_ = 46.06, *p* < 0.0001) rats. In both LA and HA rats, ν_c_ was significantly decreased by ATO and increased by MPH, while AMPH significantly increased ν_c_ only in HA rats, not in LA rats.

**Table 2:** Drug effects on TVA- and standard 5CSRTT parameters (Latin-square 1)

	Atomoxetine			Methylphenidate			Amphetamine		
	All	LA	HA	All	LA	HA	All	LA	HA
ν_c_	*t*_618_ = − 17.58 ***p* < 0.0001**	*t*_186_ = − 14.66 ***p* < 0.0001**	*t*_186_ = − 4.78 ***p* < 0.0001**	*t*_618_ = 6.40 ***p* < 0.0001**	*t*_186_ = 3.80 ***p* = 0.0006**	*t*_186_ = 5.75 ***p* = 0.0001**	*t*_618_ = 2.68 ***p* = 0.021**	*t*_186_ = − 0.90 *p* = 0.67	*t*_186_ = 4.56 ***p* < 0.0001**
ν_i_	*t*_618_ = − 8.59 ***p* < 0.0001**	*t*_186_ = − 5.48 ***p* < 0.0001**	*t*_186_ = − 2.43 ***p* = 0.044**	*t*_618_ = 7.44 ***p* < 0.0001**	*t*_186_ = 7.65 ***p* < 0.0001**	*t*_186_ = 3.04 ***p* = 0.0078**	*t*_618_ = 2.52 ***p* = 0.033**	*t*_186_ = 0.20 *p* = 0.98	*t*_186_ = 1.06 *p* = 0.57
*p*_Guess_	*t*_618_ = − 36.14 ***p* < 0.0001**	*t*_186_ = − 23.28 ***p* < 0.0001**	*t*_186_ = − 17.66 ***p* < 0.0001**	*t*_618_ = 8.71 ***p* < 0.0001**	*t*_186_ = 5.18 ***p* < 0.0001**	*t*_186_ = 5.49 ***p* < 0.0001**	*t*_618_ = 6.57 ***p* < 0.0001**	*t*_186_ = 3.48 ***p* = 0.0018**	*t*_186_ = 8.53 ***p* < 0.0001**
Accuracy	*t*_434_ = 1.09 *p* = 0.56	*t*_130_ = 0.61 *p* = 0.85	*t*_130_ = 0.34 *p* = 0.95	*t*_434_ = − 0.64 *p* = 0.83	*t*_130_ = − 0.71 *p* = 0.79	*t*_130_ = 0.31 *p* = 0.96	*t*_434_ = − 0.63 *p* = 0.83	*t*_130_ = − 0.55 *p* = 0.88	*t*_130_ = 0.10 *p* = 1.0
Correct responses	*t*_434_ = − 8.12 ***p* < 0.0001**	*t*_130_ = − 5.78 ***p* < 0.0001**	*t*_130_ = − 2.07 *p* = 0.10	*t*_434_ = 0.58 *p* = 0.86	*t*_130_ = 0.52 *p* = 0.89	*t*_130_ = 0.91 *p* = 0.67	*t*_434_ = 0.50 *p* = 0.90	*t*_130_ = 0.44 *p* = 0.92	*t*_130_ = 0.98 *p* = 0.63
Omissions	*t*_434_ = 15.13 ***p* < 0.0001**	*t*_130_ = 8.86 ***p* < 0.0001**	*t*_130_ = 6.73 ***p* < 0.0001**	*t*_434_ = − 4.42 ***p* < 0.0001**	*t*_130_ = − 2.35 *p* = 0.055	*t*_130_ = − 2.79 ***p* = 0.017**	*t*_434_ = − 2.95 ***p* < 0.0096**	*t*_130_ = − 0.85 *p* = 0.71	*t*_130_ = − 3.74 ***p* = 0.0008**
Latency correct	*t*_433_ = 7.94 ***p* < 0.0001**	*t*_129_ = 3.76 ***p* = 0.0008**	*t*_130_ = 3.59 ***p* = 0.0014**	*t*_433_ = − 3.81 ***p* = 0.0005**	*t*_129_ = − 3.29 ***p* = 0.0038**	*t*_130_ = − 0.79 *p* = 0.75	*t*_433_ = − 5.17 ***p* < 0.0001**	*t*_130_ = − 2.53 ***p* = 0.035**	*t*_130_ = − 2.20 *p* = 0.079
Latency collect	*t*_433_ = 11.34 ***p* < 0.0001**	*t*_129_ = 4.70 ***p* < 0.0001**	*t*_130_ = 6.17 ***p* < 0.0001**	*t*_433_ = -0.34 *p* = 0.95	*t*_129_ = 0.089 *p* = 1.0	*t*_130_ = 0.45 *p* = 0.92	*t*_433_ = -5.88 ***p* < 0.0001**	*t*_130_ = -3.47 ***p* = 0.0021**	*t*_130_ = -2.39 ***p* = 0.050**
Premature responses	*t*_618_ = − 16.40 ***p* < 0.0001**	*t*_186_ = − 8.76 ***p* < 0.0001**	*t*_186_ = − 5.00 ***p* < 0.0001**	*t*_618_ = 15.18 ***p* < 0.0001**	*t*_186_ = 10.59 ***p* < 0.0001**	*t*_186_ = 4.49 ***p* < 0.0001**	*t*_618_ = 13.56 ***p* < 0.0001**	*t*_186_ = 10.74 ***p* < 0.0001**	*t*_186_ = 4.98 ***p* < 0.0001**

Pairwise comparisons between drug and vehicle treatments (Dunnett’s multiple comparisons corrected). Visual processing speed (Hz) for correct (**ν**_**c**_) and incorrect (**ν**_**i**_) responses. *p*_Guess_, probability of guessing. *p*, calculated probability (*p*-value; significance indicated in bold). *LA*, low-attention rats; *HA*, high-attention rats

For ν_i_ (Fig. 1D and Table 2), we found a significant effect of drugs overall (*F*_3, 618_ = 90.02, *p* < 0.0001); ATO significantly decreased ν_i_, while both MPH and AMPH significantly increased ν_i_. We found a significant drug × phenotype interaction (*F*_3, 372_ = 23.23, *p* < 0.0001) and significant main effect of drug (*F*_3, 372_ = 66.07, *p* < 0.0001), but not of phenotype. Significant drug effects were present in LA (*F*_3, 186_ = 58.05, *p* < 0.0001) and HA (*F*_3, 186_ = 10.37, *p* < 0.0001) rats. In both LA and HA rats, ν_i_ was significantly decreased by ATO and increased by MPH. AMPH did not affect ν_i_ in neither LA nor HA rats.

For *p*_g_ (Fig. 1B and Table 2), we found a significant effect of drugs overall (*F*_3, 618_ = 877.31, *p* < 0.0001); ATO significantly decreased, while MPH and AMPH significantly increased, the probability of guessing. We found a significant drug × phenotype interaction (*F*_3, 372_ = 9.48, *p* < 0.0001), and a significant main effect of drug (*F*_3, 372_ = 617.51, *p* < 0.0001), but not of phenotype. Significant drug effects were present both in LA (*F*_3, 186_ = 351.76, *p* < 0.0001) and HA (*F*_3, 186_ = 274.35, *p* < 0.0001) rats. In both LA and HA rats, guessing probability was decreased by ATO and increased by MPH and AMPH.

Some drugs affected the associations between TVA-modelled and standard parameters (Figs. S6-S7 and Table S1). In short, ATO induced a positive correlation between ν_c_ and errors committed and removed correlation with omissions, while producing an association between higher ν_i_ and reduced inhibitory control. AMPH treatment produced an association of higher ν_c_ with higher accuracy and fewer errors. MPH did not affect correlations.

### Effects of atipamezole and phenyphrine on modelled TVA parameters

For ν_c_ (Fig. 1C and Table 3), we found a significant effect of drugs overall (*F*_2, 431.36_ = 65.80, *p* < 0.0001); ν_c_ was significantly decreased by PHEN and increased by ATI. We found no drug × phenotype interaction or main effect of phenotype, but there was a significant main effect of drugs (*F*_2, 249.37_ = 57.61, *p* < 0.0001).

**Table 3:** Drug effects on TVA- and standard 5CSRTT parameters (Latin-square 2)

	Phenylephrine			Atipamezole		
	All	LA	HA	All	LA	HA
ν_c_	*t*_432_ = − 5.26 ***p* < 0.0001**	*t*_111_ = − 6.03 ***p* < 0.0001**	*t*_138_ = − 2.37 ***p* = 0.037**	*t*_431_ = 6.29 ***p* < 0.0001**	*t*_111_ = 8.68 ***p* < 0.0001**	*t*_138_ = 3.26 ***p* = 0.0028**
ν_i_	*t*_432_ = − 6.74 ***p* < 0.0001**	*t*_111_ = − 6.81 ***p* < 0.0001**	*t*_138_ = − 2.70 ***p* = 0.015**	*t*_431_ = 6.06 ***p* < 0.0001**	*t*_111_ = 9.53 ***p* < 0.0001**	*t*_138_ = 3.74 ***p* = 0.0005**
*p*_Guess_	*t*_431_ = − 5.43 ***p* < 0.0001**	*t*_111_ = − 3.92 ***p* = 0.0003**	*t*_138_ = − 5.80 ***p* < 0.0001**	*t*_431_ = 9.30 ***p* < 0.0001**	*t*_111_ = 7.17 ***p* < 0.0001**	*t*_138_ = 7.62 ***p* < 0.0001**
Accuracy	*t*_305_ = 0.16 *p* = 0.97	*t*_80.9_ = 0.58 *p* = 0.78	*t*_96_ = 0.16 *p* = 0.97	*t*_301_ = 0.07 *p* = 0.99	*t*_77.1_ = 0.34 *p* = 0.91	*t*_96_ = -0.34 *p* = 0.91
Correct responses	*t*_304_ = − 2.79 ***p* = 0.011**	*t*_78.6_ = − 3.50 ***p* = 0.0015**	*t*_96_ = − 1.08 *p* = 0.46	*t*_301_ = 1.52 *p* = 0.23	*t*_77_ = 1.09 *p* = 0.45	*t*_96_ = 0.93 *p* = 0.55
Omissions	*t*_302_ = 2.75 ***p* = 0.012**	*t*_77.7_ = 0.32 *p* = 0.91	*t*_96_ = 3.41 ***p* = 0.0019**	*t*_301_ = − 5.22 ***p* < 0.0001**	*t*_77_ = − 5.18 ***p* < 0.0001**	*t*_96_ = − 3.54 ***p* = 0.0012**
Premature responses	*t*_432_ = − 3.16 ***p* = 0.0033**	*t*_112_ = − 0.31 *p* = 0.92	*t*_111_ = − 5.61 ***p* < 0.0001**	*t*_431_ = 10.02 ***p* < 0.0001**	*t*_111_ = 8.02 ***p* < 0.0001**	*t*_138_ = 7.07 ***p* < 0.0001**
Latency correct	*t*_303_ = 2.53 ***p* = 0.023**	*t*_77.9_ = 0.50 *p* = 0.82	*t*_96_ = 1.47 *p* = 0.26	*t*_301_ = − 3.73 ***p* = 0.0005**	*t*_77_ = − 3.83 ***p* = 0.0005**	*t*_96_ = − 1.41 *p* = 0.28
Latency collect	*t*_301_ = 4.98 ***p* < 0.0001**	*t*_77.4_ = 1.53 *p* = 0.23	*t*_96_ = 3.77 ***p* = 0.0006**	*t*_301_ = − 0.83 *p* = 0.61	*t*_77_ = − 1.36 *p* = 0.31	*t*_96_ = − 2.06 *p* = 0.085

Pairwise comparisons between drug and vehicle treatments (Dunnett’s multiple comparisons corrected). Visual processing speed (Hz) for correct (**ν**_**c**_) and incorrect (**ν**_**i**_) responses. *p*_Guess_, probability of guessing. *p*, calculated probability (*p*-value﻿; significance indicated in bold). *LA*, low-attention rats; HA, high-attention rats

For ν_i_ (Fig. 1D and Table 3), we found a significant effect of drugs overall (*F*_2, 431.68_ = 80.68, *p* < 0.0001); ν_i_ was significantly decreased by PHEN and increased by ATI. We found a significant drug × phenotype interaction (*F*_2, 249.55_ = 24.75, *p* < 0.0001) and significant main effect of drug (*F*_2, 249.55_ = 125.25, *p* < 0.0001), but not of phenotype. Significant drug effects were present in LA (*F*_2, 111.27_ = 126.56, *p* < 0.0001) and HA (*F*_2, 138_ = 20.90, *p* < 0.0001) rats. Both in LA and HA rats, ν_i_ was significantly decreased by PHEN and increased by ATI.

For guessing probability (Fig. 1B and Table 3), we found a significant effect of drugs overall (*F*_2, 431.21_ = 109.13, *p* < 0.0001); *p*_g_ was significantly decreased by PHEN and increased by ATI. We found a significant main effect of drug (*F*_2, 249.57_ = 142.26, *p* < 0.0001), but no drug × phenotype interaction or main effect of phenotype.

Some drugs affected the associations between visual processing speed and other parameters (Figs. S6-S7 and Table S1). Like AMPH, ATI induced a positive correlation between ν_c_ and accuracy. ATI also produced a positive correlation between ν_c_ (and ν_i_) and reward magazine perseveration. PHEN did not significantly change correlations.

### Effects of atomoxetine, methylphenidate and amphetamine on standard vSD-5CSRTT parameters

**Fig. 2:**
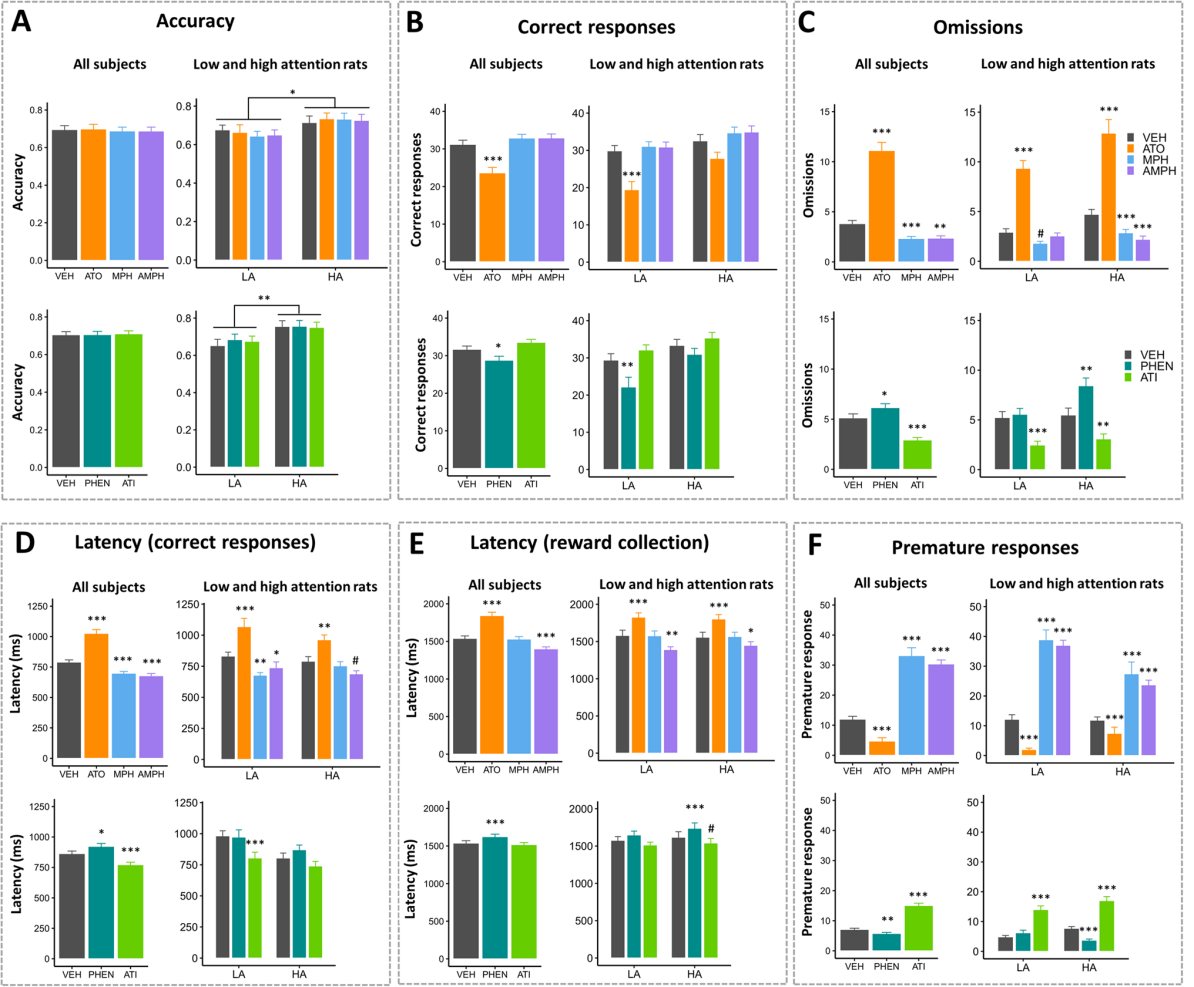
Effects of stimulant and non-stimulant drugs on standard 5CSRTT parameters. **A**–**D** presents results for Latin-square 1 (LS1, top panels) and Latin-square 2 (LS2, bottom panels) with standard 5CSRTT parameters accuracy (**A**), correct responses (**B**), omissions (**C**), latency to respond correctly (**D**), reward collection latency (**E**) and premature responses (**F**). LA, low-attention rats; HA, high-attention rats; ATO, atomoxetine; MPH, methylphenidate; AMPH, amphetamine; PHEN, phenylephrine; ATI, atimpamezole. Results are represented as mean ± SEM; ***p < 0.001; **p < 0.01; *p < 0.05; #*p* < 0.1

No drugs affected accuracy (Fig. 2A and Table 2), but there was a main effect of phenotype (*F*_1, 12_ = 7.52, *p* = 0.018). This confirms that LA rats had significantly lower accuracy than HA rats, irrespective of treatment.

Drug treatment significantly affected correct responses (Fig. 2B and Table 2) (*F*_3, 434_ = 36.08, *p* < 0.0001); ATO significantly reduced correct responses, while MPH and AMPH had no effects. We found a significant drug × phenotype interaction (*F*_3, 260_ = 3.79, *p* = 0.011) as well as a main effect of drug (*F*_3, 260_ = 20.58, *p* < 0.001) and a trending effect of phenotype (*F*_1, 12_ = 4.56, *p* = 0.054). Significant drug effects were present in LA (*F*_3, 130_ = 18.71, *p* < 0.0001) and HA (*F*_3, 130_ = 4.05, *p* = 0.0086) rats; ATO decreased number of correct responses in LA rats, not HA, rats.

For omissions (Fig. 2C and Table 2), we found a significant effect of drugs overall (*F*_3, 434_ = 161.34, *p* < 0.0001), where ATO significantly increased number of omissions, while MPH and AMPH significantly decreased it. We found a trending drug × phenotype interaction (*F*_3, 260_ = 2.36, *p* = 0.072) and a significant main effect of drugs (*F*_3, 260_ = 92.06, *p* < 0.0001), but not of phenotype. Significant drug effects were present in LA (*F*_3, 130_ = 51.21, *p* < 0.0001) and HA (*F*_3, 130_ = 44.71, *p* < 0.0001) rats; ATO increased omissions in LA and HA rats, while MPH decreased omissions in HA rats and trended towards doing so in LA rats. On the other hand, AMPH only decreased omissions in HA rats, without affecting LA rats.

For latency to respond correctly (Fig. 2D and Table 2), we found a significant effect of drugs overall (*F*_3, 433.01_ = 69.12, *p* < 0.0001); ATO significantly prolonged correct latency, while MPH and AMPH significantly speeded it. When investigating for phenotype dependency, we found a significant main effect of drug (*F*_3, 259.02_ = 30.84, *p* < 0.0001), but no drug × phenotype interaction and no main effect of phenotype.

For reward collection latency (Fig. 2E and Table 2), we found a significant effect of drugs overall (*F*_3, 433_ = 104.18, *p* < 0.0001); collection latency was significantly lengthened by ATO and shortened by AMPH, while MPH had no effect. When investigating for phenotype dependency, we found a significant main effect of drug (*F*_3, 259_ = 47.20, *p* < 0.0001), but no drug × phenotype interaction and no main effect of phenotype.

For premature reponses (Fig. 2F and Table 2), we found a significant effect of drugs overall (*F*_3, 618_ = 430, *p* < 0.0001); ATO significantly decreased premature responses, while MPH and AMPH significantly increased prematures. We found a significant drug × phenotype interaction (*F*_3, 372_ = 14.91, *p* < 0.0001) and significant main effect of drug (*F*_3, 372_ = 188.00, *p* < 0.0001), but not of phenotype. Significant drug effects were present in LA (*F*_3, 186_ = 176.5, *p* < 0.0001) and HA (*F*_3, 186_ = 43.37, *p* < 0.0001) rats. In both LA and HA rats, premature responses were significantly decreased by ATO and increased by MPH and AMPH.

### Effects of atipamezole and phenylephrine on standard vSD-5CSRTT parameters

No drugs affected accuracy (Fig. 2A and Table 3), but there was a main effect of phenotype (*F*_1, 10.5_ = 12.97, *p* = 0.0045), confirming that LA rats had significantly lower accuracy than HA rats irrespective of treatment.

For correct responses (Fig. 2B and Table 3), we found a significant effect of drugs overall (*F*_2, 302.7_ = 9.42, *p* < 0.0001); PHEN significantly reduced number of correct responses, while ATI had no effect. We found a significant drug × phenotype interaction (*F*_2, 175.01_ = 3.48, *p* = 0.033) as well as a main effect of drug (*F*_2, 175.01_ = 12.55, *p* < 0.001) and phenotype (*F*_1, 11.18_ = 6.03, *p* = 0.032). Significant drug effects were present in LA rats (*F*_2, 78.15_ = 11.00, *p* < 0.0001), but not HA rats (*F*_2, 96_ = 2.02, *p* = 0.14); PHEN decreased the number of correct responses in LA rats, without affecting HA rats.

For omissions (Fig. 2C and Table 3), we found a significant effect of drugs overall (*F*_2, 301.67_ = 32.25, *p* < 0.0001). PHEN significantly increased number of omissions, while ATI significantly decreased it. We found a trending drug × phenotype interaction (*F*_2, 173.75_ = 2.90, *p* = 0.058) and a significant main effect of drugs (*F*_2, 173.75_ = 36.84, *p* < 0.0001), but not of phenotype. Significant drug effects were present in LA rats (*F*_2, 77.48_ = 18.22, *p* < 0.0001) and HA rats (*F*_2, 96_ = 24.14, *p* < 0.0001); PHEN increased omissions in HA rats, not LA rats, while ATI decreased omissions both in LA and HA rats.

For correct response latency (Fig. 2D and Table 3), we found a significant effect of drugs overall (*F*_2, 300.54_ = 19.53, *p* < 0.0001); ATI significantly decreased response latency, while PHEN increased it. When investigating phenotype dependency, we found a significant main effect of drug (*F*_2, 173.51_ = 12.39, *p* < 0.0001), a trending main effect of phenotype (*F*_1, 10.23_ = 3.63, *p* = 0.085), but no drug × phenotype interaction.

For reward collection latency (Fig. 2E and Table 3), we found a significant effect of drugs overall (*F*_2, 301.1_ = 19.50, *p* < 0.0001); PHEN significantly slowed collection latency, while ATI had no effect. When investigating for phenotype dependency, we found a significant main effect of drug (*F*_2, 173.14_ = 17.04, *p* < 0.0001), but no drug × phenotype interaction and no main effect of phenotype.

For premature reponses (Fig. 2F and Table 3), we found a significant effect of drugs overall (*F*_2, 431.49_ = 93.27, *p* < 0.0001), with premature responses being significantly decreased by PHEN and increased by ATI. We found a significant drug × phenotype interaction (*F*_2, 249.57_ = 6.80, *p* = 0.0013) and a significant main effect of drug (*F*_2, 249.57_ = 110.00, *p* < 0.0001), but not of phenotype. Significant drug effects were present in LA (*F*_2, 111.33_ = 42.7, *p* < 0.0001) and HA (*F*_2, 138_ = 80.66, *p* < 0.0001) rats. Premature responses were decreased by PHEN in HA, not in LA, and were increased by ATI in both LA and HA rats.

### Results summary

Main results are summarised in Table 4.

**Table 4:** Results summary

	Atomoxetine			Methylphenidate			Amphetamine			Phenylephrine			Atipamezole		
	All	LA	HA	All	LA	HA	All	LA	HA	All	LA	HA	All	LA	HA
ν_c_	↓	↓	↓	↑	↑	↑	↑	—	↑	↓	↓	↓	↑	↑	↑
ν_i_	↓	↓	↓	↑	↑	↑	↑	—	—	↓	↓	↓	↑	↑	↑
*p*_Guess_	↓	↓	↓	↑	↑	↑	↑	↑	↑	↓	↓	↓	↑	↑	↑
Accuracy	—	—	—	—	—	—	—	—	—	—	—	—	—	—	—
Correct responses	↓	↓	(↓)	—	—	—	—	—	—	↓	↓	—	—	—	—
Omissions	↑	↑	↑	↓	(↓)	↓	↓	—	↓	↑	—	↑	↓	↓	↓
Latency correct	↑	↑	↑	↓	↓	—	↓	↓	(↓)	↓	—	↓	↑	↑	↑
Latency collect	↑	↑	↑	—	—	—	↓	↓	↓	↑	—	—	↓	↓	—
Premature responses	↓	↓	↓	↑	↑	↑	↑	↑	↑	↑	—	↑	—	—	(↓)

## Discussion

To understand whether stimulant and non-stimulant drugs specifically affect visual attentional processing, we adapted the human TVA model (Bundesen 1990; Bundesen and Harms 1999; Bundesen et al. 2005) to the rat 5CSRTT. We administered drugs relevant for ADHD pharmacological therapies, i.e. AMPH, MPH and ATO, as well as the relatively selective noradrenergic agents, ATI and PHEN targeting the α2- and α1-adrenoceptors, respectively. While no drugs affected accuracy, dissociable effects were observed on TVA-modelled visual processing speed. ATO and PHEN surprisingly slowed, whereas ATI and MPH speeded up visual processing, both for correct and incorrect decisions. Thus, in the present study, ATO produced attentional deficits possibly due to slowed visual processing. In contrast, AMPH selectively improved visual processing for correct, not incorrect, decisions in HA rats, reflecting improved attention in high performers—surprisingly, without affecting low performers. Overall, this suggests catecholaminergic modulation to be involved in visual attentional processing.

### Effects of stimulant drugs on attention; MPH versus AMPH

That AMPH and MPH did not affect accuracy is generally consistent with previous 5CSRTT studies in healthy rodents treated with comparable (low to moderate) doses of MPH (Navarra et al. 2008; Milstein et al. 2010; Fernando et al. 2012; Pattij et al. 2012; Hauser et al. 2017) and AMPH (Cole and Robbins 1987; Harrison et al. 1999; Van Gaalen et al. 2006; Loos et al. 2010; Balachandran et al. 2018; Higgins et al. 2020). Although a few studies have reported these drugs to improve accuracy during increased task demand and/or in low-attention rats (Koffarnus and Katz 2011; Robinson 2012; Caballero-Puntiverio et al. 2017; Toschi et al. 2021) as well as in high-impulsive rats (Caprioli et al. 2015), we did not observe any effects on accuracy in LA rats. Studies using other rodent attentional tasks have reported beneficial attentional effects of comparable doses of AMPH or MPH (Berridge et al. 2006, 2012; Tomlinson et al. 2014; Turner and Burne 2016; Navarra et al. 2017; MacQueen et al. 2018; Caballero-Puntiverio et al. 2019; Young et al. 2020) as well as in a genetic ADHD-like mouse model (Nilsson et al. 2018) and prefrontal cortex lesioned animals (Chudasama et al. 2005), although not in all studies (Ding et al. 2018; Caballero-Puntiverio et al. 2020). Thus, while results have been inconsistent, low doses of stimulant drugs potentially facilitate certain attentional processes, which may not be fully captured by standard 5CSRTT attentional parameters such as accuracy. Supporting this, AMPH and MPH did induce fewer omissions and faster responding, which may reflect improved attention (Lezak et al. 2012), general arousal (e.g. Rapoport et al. 1980; Berridge 2006) or reduced fatigue (Choi and Raymer 2019).

To understand whether MPH and AMPH specifically affect visual attentional processing, we applied the TVA model. Human TVA modelling has shown MPH to improve visual processing speed in participants with poor baseline attention (Finke et al. 2010). In our study, MPH increased visual processing speed both for correct and incorrect responses in LA and HA rats, indicating a more general arousal effect rather than a specific attentional effect. On the other hand, AMPH did not affect visual processing in LA rats, but selectively improved attention in HA rats, as visual processing speed was enhanced for correct responses without affecting it for incorrect responses. Correspondingly, omissions were also reduced by AMPH only in HA rats. Further supporting an AMPH-induced change in attentional capacity is that AMPH produced a positive correlation between ν_c_ and accuracy; thus, higher accuracy was associated with higher ν_c_—this was not the case for MPH (or VEH) treated rats. Thus, in our study, AMPH improved attentional capacity in high performers specifically indicating that AMPH treatment can improve visual attentional processing, although not necessarily in low-attention individuals as might have been expected given its efficacy in treating ADHD.

While attentional effects of stimulants have been variable in previous studies, it is a consistent finding in rodent 5CSRTT studies that both AMPH and MPH induce impulsivity, as well as hyperactivity (e.g. Cole and Robbins 1987; Harrison et al. 1999; Pattij et al. 2007; Navarra et al. 2008; Baarendse and Vanderschuren 2012; Higgins et al. 2020; Toschi et al. 2021), consistent with the present study.

Altogether, the stimulant-induced effects indicate heightened arousal or overall behavioural activation, possibly due to enhanced motivation; which is also supported by an increased willingness to ‘guess’ under uncertainty, when no information is available. Worth noting, in contrast to AMPH-induced improving effects on omissions and visual attentional processing, the speeding effect on reaction times was evident only in LA rats, indicating differential underlying neural mechanisms. Thus, AMPH-induced improved attentional processing, and decreased omissions are not directly associated with faster responding, indicating that decreased omissions may reflect attentional engagement and task motivation, while faster responding may be more associated with locomotor activation, i.e. hyperactivity. This is further supported by the fact that ν_c_ did not correlate with latencies, but rather it correlated negatively with omissions and positively with correct responses.

Our observation that AMPH facilitates attentional processing adds to previous studies reporting pro-attentional effects of psychostimulants in healthy humans (e.g. metanalyses (Marraccini et al. 2016)) and ADHD patients (e.g. Losier et al. 1996; Faraone and Biederman 2002; Faraone and Buitelaar 2010). That AMPH has a higher degree of pro-attentional effects than MPH supports a meta-analysis of human studies arriving at the same conclusion (Faraone and Buitelaar 2010). Furthermore, it is in line with a recent study showing AMPH, not MPH or ATO, to improve visual processing speed in a human continuous performance task (CPT) with TVA modelling—a novel CombiTVA paradigm (Lansner 2022). These results highlight the importance of refining the study of stimulant drug effects on attention with tools, such as the TVA model, that can measure attentional effects previously reported in humans, but not directly captured by standard 5CSRTT parameters.

### Effects of non-stimulant versus stimulant drugs on attention: ATO versus MPH/AMPH

For nearly all parameters, ATO-induced behavioural effects contrasted with MPH- and AMPH-induced effects. ATO slowed visual processing, as modelled by TVA, both for correct and incorrect responses, indicating diminished attentional capacity. This was not reflected in accuracy though, which was, like MPH and AMPH, not affected by ATO, in line with previous rodent attention studies using similar doses of ATO (Blondeau and Dellu-Hagedorn 2007; Robinson et al. 2008; Tsutsui-Kimura et al. 2009; Fernando et al. 2012; Sun et al. 2012; Pillidge et al. 2014; Ding et al. 2018; Higgins et al. 2020). However, some studies have shown ATO-induced attentional improvement during vSD attentional challenge in rats and mice (Caballero-Puntiverio et al. 2019, 2020; Callahan et al. 2019) or in poorly performing rats (Robinson 2012; Tomlinson et al. 2014) and, on the other hand, attentional impairment in highly performing rats (Tomlinson et al. 2014) and under a variable ITI challenge (Higgins et al. 2020; Toschi et al. 2021).

In humans, only a few studies have assessed attentional effects of acute ATO. ATO improves rapid visual information processing (Crockett et al. 2010), but has no effect on attentional performance in a stop-signal reaction time task (Chamberlain et al. 2007) or in a recent human CombiTVA study, which also showed reduced short-term memory capacity after ATO (Lansner 2022). However, a positive association has been found between the dopamine beta-hydroxylase genotype (responsible for NA synthesis) and sustained attention in human TVA-modelled CPT (Shalev et al. 2019). Thus, acute ATO treatment has shown inconsistent effects on attentional parameters depending on attentional load, task, baseline performance and presumably also dose.

The present study shows detrimental effects of ATO on visual attentional processing. However, the observed ATO-induced slowed visual processing may not specifically indicate poor attention, but could be secondary to a general behavioural slowing as ν_c_ and ν_i_ are both slowed and accompanied by slowed motor responding and reward collection. Accordingly, ATO also generally reduced correct, incorrect and premature reponses as well as increased omissions and, consequently, a reduced willingness to ‘guess’ (i.e. random responses). Thus, in addition to possibly reflecting inattentiveness, general behavioural slowing may also reflect lack of motivation and general hypoactivity (i.e. mild sedation). ATO-induced slowed reaction times has been reported in previous rodent 5CSRTT studies (Blondeau and Dellu-Hagedorn 2007; Bari et al. 2009; Jentsch et al. 2009; Baarendse and Vanderschuren 2012; Fernando et al. 2012; Robinson 2012; Sun et al. 2012; Benn and Robinson 2017; Ding et al. 2018; Caballero-Puntiverio et al. 2019), although not in others (Robinson et al. 2008; Tsutsui-Kimura et al. 2009; Koffarnus and Katz 2011; Paterson et al. 2011, 2012; Pillidge et al. 2014; Liu et al. 2015). In humans, ATO generally does not affect reaction times (Shang and Gau 2012; Ni et al. 2013, 2016; Bédard et al. 2015). In fact, some studies in humans contrast rodent studies, reporting ATO to actually shorten reaction times (Gau and Shang 2010; Wehmeier et al. 2011, 2012; Kratz et al. 2012; Fan et al. 2017). That ATO may decrease motivation is supported by the slowed reward collection by ATO in the present study and previous 5CSRTT studies in rodents (Navarra et al. 2008; Pillidge et al. 2014), as well as ATO-induced dimished motivation for effort-demanding reward collection in a progressive ratio test independent of locomotor activity (Higgins et al. 2020). Consistently, NA is an appetite-suppressant in rodents (Rinaman 2011; Roman et al. 2016) and humans with ADHD (Hah and Chang 2005; Kratochvil et al. 2011; Walker et al. 2015). Taken together, slowing effects of ATO in the present study may be due to hypoactivity (i.e. mild sedation) in conjunction with decreased motivation for reward.

The opposing effects of MPH/AMPH and ATO are consistent with dissociable behavioural effects on the 5CSRTT of ATO and MPH in striatal regions (Economidou et al. 2012), and also of increased DA stimulating motivation (Achterberg et al. 2016; Yohn et al. 2016a) and increased NA diminishing motivation, possibly, to some degree, through ATO-induced serotonergic modulation (Gallezot et al. 2011; Mathes et al. 2013; Rosenberg et al. 2013; Ding et al. 2014; Yohn et al. 2016b, a)—although presumably not in the PFC (Bymaster et al. 2002). Furthermore, similar to MPH and AMPH, ATO increases extracellular NA and DA levels in the prefrontal cortex (Bymaster et al. 2002). However, in contrast to stimulants, ATO does not affect (Bymaster et al. 2002; Heal et al. 2009) or may even decrease (Yohn et al. 2016a), DA release in striatal regions, which may explain why ATO reduces response rate and speed, as opposed to stimulants. Additonally, via its actions on NA mediated by alpha-1 receptors, ATO can also indirectly increase prefrontal cortical acetylcholine at 1 mg/kg, which may thus contribute to ATO’s working memory enhancing effects (Tzavara et al. 2006). Therefore, although ATO is a highly specific NET inhibitor, some of its effects may ultimately be mediated by its indirect actions on other neurotransmitters, although it seems unlikely that any pro-cholinergic actions of ATO would produce slowed visual processing as seen here. Moreover, Bari et al. (2011) provided pharmacological evidence that the ameliorative effects on impulsive responding produced by intra-PFC ATO were mediated by noradrenergic rather than dopaminergic mechanisms.

Our results suggest catecholaminergic modulation to be implicated in visual attentional processing, and that it may play a complementary role to the cholinergic system in attention, as we previously showed anti-cholinergic treatment to reduce TVA-modelled visual processing speed (Fitzpatrick et al. 2017). The slowing effects of ATO actually are similar to some anti-cholinergic effects seen in the mouse TVA-5CSRTT, where scopolamine slowed visual processing as well as reaction times and reward collection, while also increasing omissions. However, in contrast to ATO, scopolamine increased premature responses, indicating separate underlying actions.

### Involvement of α1- and α2-adrenoceptors in attention

As ATO increases extracellular NA globally, it was relevant to investigate the role of specific adrenoceptor subtypes. Previous studies have indicated an attentional role for the high-affinity and abundant α1-adrenoceptors (Berridge 2006; Spencer et al. 2012), which, in the present study, was activated by PHEN. That PHEN had similar behavioural effects to ATO across nearly all parameters, including slowed visual processing, suggests that, at least partly, ATO’s effects are mediated via α1-adrenoceptor activation, but this was not directly tested in this study. The lack of PHEN-induced effects on accuracy, and its slowing effect on visual processing, somewhat contradicts previous studies claiming that improvements on attention (in rats) following dopamine D3 agonist- (Marshall et al. 2019) or low-dose MPH administration (Berridge et al. 2006, 2012; Navarra et al. 2017) were dependent on activation of the α1-adrenoceptor (Berridge et al. 2012). Similar claims of a pro-cognitive effect of activating the α1-adrenoceptor were brought forward by studies showing that the putative α1-adrenoceptor agonist, St-587, improves accuracy in the 5CSRTT with shortened SD (Puumala et al. 1997) and that α1-adrenoceptor antagonism impairs 5CSRTT accuracy (Puumala et al. 1997) and go accuracy in a rat stop-signal reaction time task (Bari and Robbins 2013) while slowing responding (Hahn and Stolerman 2005; Bari and Robbins 2013). Thus, our data does not provide evidence for the claim that α1-adrenoceptor activation increases vigilance as previously suggested (Sirviö and MacDonald 1999); instead, it induced a general behavioural hypoactivity, similar to ATO. Although we had chosen a dose of PHEN previously shown not to slow rats (Pattij et al. 2012), we cannot rule out that we would have seen potential beneficial effects had we tested lower doses of PHEN.

Cortical NA depletion does not affect choice accuracy in rat 5CSRTT (Ruotsalainen et al. 1997), but impairs performance when attentional demand is increased (Carli et al. 1983; Milstein et al. 2007; Cole and Robbins 1992). This impairment is exacerbated by α2 agonism (Milstein et al. 2007), indicating potentially benefical effects of blocking the α2-adrenoceptor. A few previous studies have shown ATI to improve 5CSRTT accuracy (Sirvio et al. 1993; Koskinen et al. 2003) or to have no effect on it (Sirviö et al. 1994). In other rodent attentional tasks, α2-adrenoceptor antagonism improves sustained attention in a stop-signal reaction time task (Bari and Robbins 2013), auditory cue detection (Brown et al. 2012) and attentional set shifting (Devauges and Sara 1990; Lapiz and Morilak 2006). However, our data do not fully support a selective pro-attentive effect of α2-adrenoceptor antagonism, rather ATI prompts a general behavioural activation similar to that of MPH, both speeding up visual processing and reaction times, while decreasing omissions and diminishing inhibitory control. This is in line with previous studies showing α2-adrenoceptor antagonism to increase locomotor activity (Niittykoski et al. 1998) and impair inhibitory response control (Sirviö et al. 1994; Ruotsalainen et al. 1997; Koskinen et al. 2003; Sun et al. 2010) in rats and healthy human subjects (Swann et al. 2005, 2013; Sun et al. 2010); effects possibly mediated by the PFC as shown in monkeys (Ma et al. 2005).

## Conclusions

Unexpectedly, we captured overall slowing effects, including impaired visual processing, of drugs increasing extracellular noradrenaline (ATO) or activating the α1-adrenoceptor (PHEN). In contrast, we found overall speeding effects of drugs enhancing both dopaminergic and noradrenergic transmission (MPH, AMPH and ATI). We conclude that, while ATO decreases impulsivity, which is presumably a significant part of its therapeutic effect in ADHD, it may also produce detrimental effects such as general behavioural slowing and diminished visual processing, at least after acute dosing. In contrast, a single low dose of amphetamine had potential pro-attentional effects by enhancing visual processing, probably due to central dopamine upregulation.

That no drugs affected accuracy, but had differential effects on visual perceptual processing speed, suggests that more temporally dynamic and detailed attentional measures, like TVA-modelled parameters, are needed to fully capture attentional effects as an addition to standard parameters, such as accuracy. Thus, these data indicate that applying TVA to 5CSRTT performance provides enhanced sensitivity to capturing attentional effects compared with standard 5CSRTT variables, both via increased attentional load and TVA modelling. This application of the TVA model to rodents further enables future translational investigations of neural mechanisms underlying visual attentional processing. The potential cross-species translational value of applying TVA modelling to the rodent 5CSRTT is exemplified by recent studies of healthy humans performing TVA-CPT with acute MPH, AMPH or ATO treatment, where AMPH was the only drug specifically improving TVA-modelled visual processing speed, and neither MPH or ATO improved visual processing speed (Lansner 2022). Nevertheless, we should also acknowledge limitations of the present findings that should be remediated by future studies: more extensive dose–response determinations are required to confirm whether the present effects observed at single doses hold over a wider range, and it will be necessary to compare the present acute actions with effects of chronic dosing, as occurs clinically in the treatment of ADHD.

## Supplementary Information

Below is the link to the electronic supplementary material.

Supplementary file1 (PDF 1462 KB)
