# Supplementary material for: Theory of visual attention (TVA) applied to rats performing the 5-choice serial reaction time task: differential effects of dopaminergic and noradrenergic manipulations
Source: Psychopharmacology (Berl). 2022 Nov 25;240(1):41–58. doi: 10.1007/s00213-022-06269-4 (PMC9816296; doi:10.1007/s00213-022-06269-4)
Supplement: Supplementary file 1 — Supplementary file1 (PDF 1462 KB) [file 213_2022_6269_MOESM1_ESM.pdf]

Supplementary material

Theory of Visual Attention (TVA) applied to rats performing the 5-choice serial reaction time task; differential effects of dopaminergic and noradrenergic manipulations.

Mona E. Hervig, Chiara Toschi, Anders Petersen, Signe Vangkilde, Ulrik Gether and Trevor W. Robbins

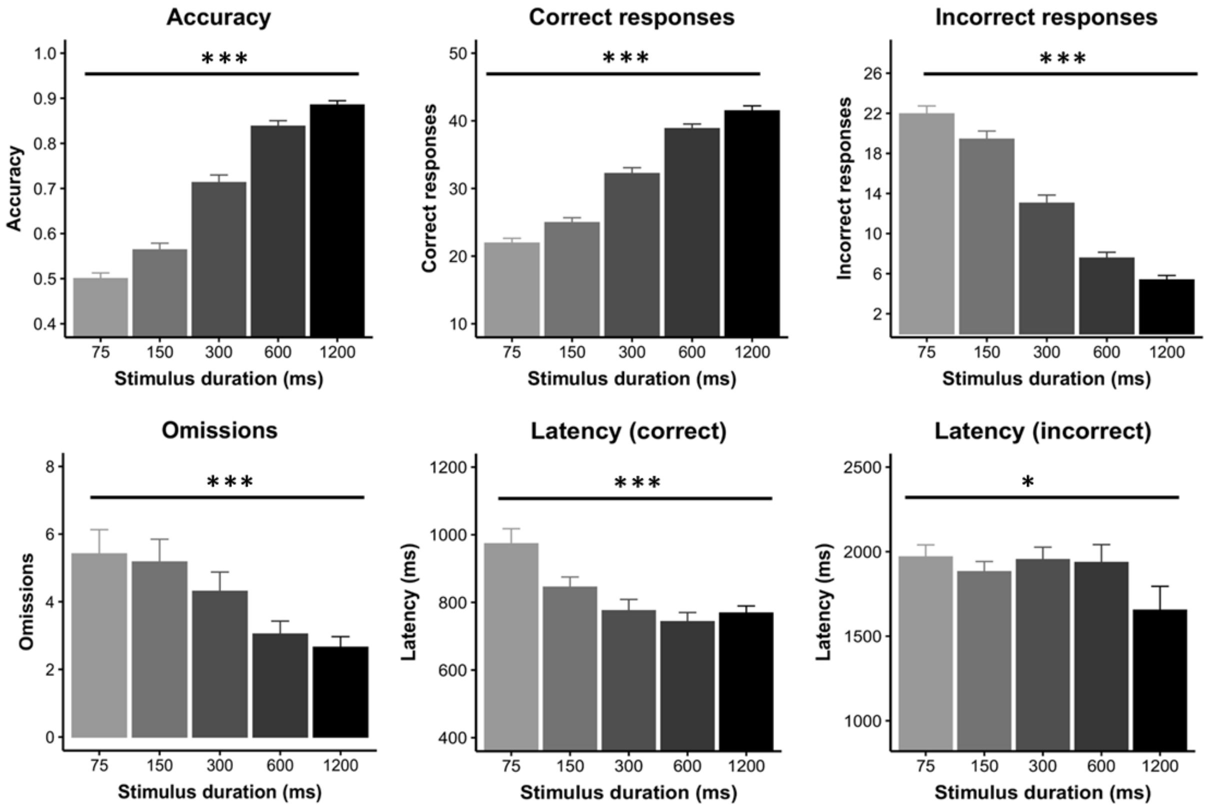

Figure S1. Effect of variable stimulus duration on 5CSRTT performance in VEH-treated rats.
We analysed parameters for vehicle treatments as a function of SDs (averaged for Latin-squares 1 and 2) to confirm the effect of vSD on performance. As expected, vSD had a significant effect on accuracy ($F_{4, 88}$ = 178.87, p < 0.0001), with improving accuracy as SDs increased (statistical significant differences between all SDs). This is also confirmed by number of correct responses ($F_{4, 88}$ = 220.25, p < 0.0001) increasing, and incorrect responses ($F_{4, 88}$ = 160, p < 0.0001) and omissions ($F_{4, 88}$ = 13.53, p < 0.0001) decreasing with increasing SDs. Also, as expected, latency of correct ($F_{4, 88}$ = 16.22, p < 0.0001) and incorrect ($F_{4, 88}$ = 3.23, p = 0.016) responses decreased with increasing SDs, while leaving reward collection latency somewhat unaffected ($F_{4, 88}$ = 2.17, p = 0.080) by increasing SDs. Furthermore, SDs correlates positively with accuracy (Pearson r = 0.83, p < 0.0001) and correct responses (Pearson r = 0.80, p < 0.0001), and negatively with incorrect responses (Pearson r = -0.81, p < 0.0001), omissions (Pearson r = -0.36, p < 0.0001), latency (correct) (Pearson r = -0.29, p = 0.001) and latency (incorrect) (Pearson r = -0.30, p = 0.001) (data not shown). Thus attentional performance overall improved with increasing SDs and was dependent on SD, as expected.

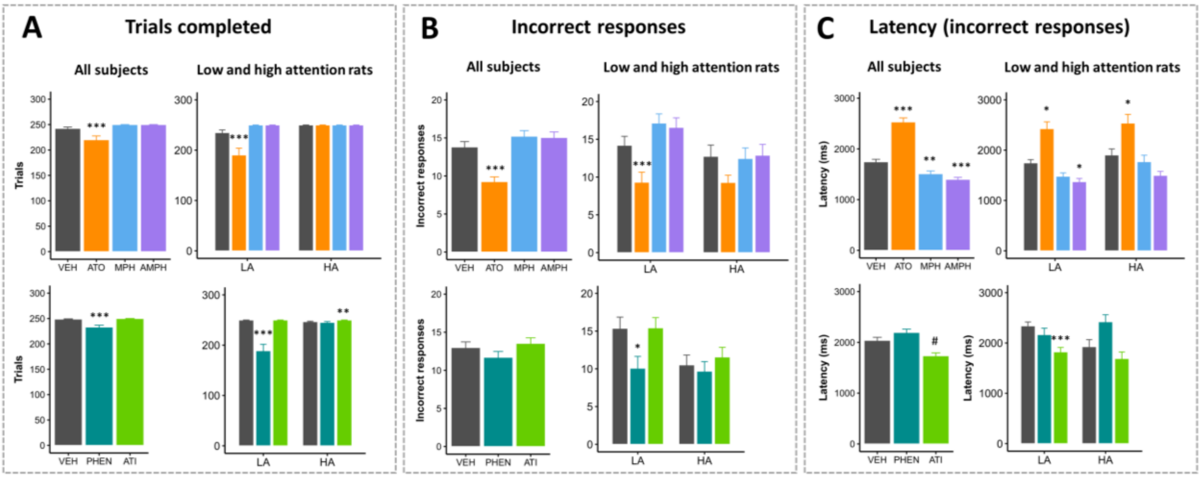

Figure S2. Standard 5CSRTT parameters trials, incorrect responses and latencies for incorrect responses. (A-C) presents results for Latin-square 1 (LS1, top panels) and Latin-square 2 (LS2, bottom panels) with standard 5CSRTT parameters trials **(A)**, incorrect responses **(B)**, latency to respond incorrectly **(C)**. LA, low attention rats; HA, high attention rats; ATO, atomoxetine; MPH, methylphenidate; AMPH, amphetamine; PHEN, phenylephrine; ATI, atimpamezole. Results are represented as mean ± SEM; ****p* < 0.001; ***p* < 0.01; **p* < 0.05; #*p* < 0.1.

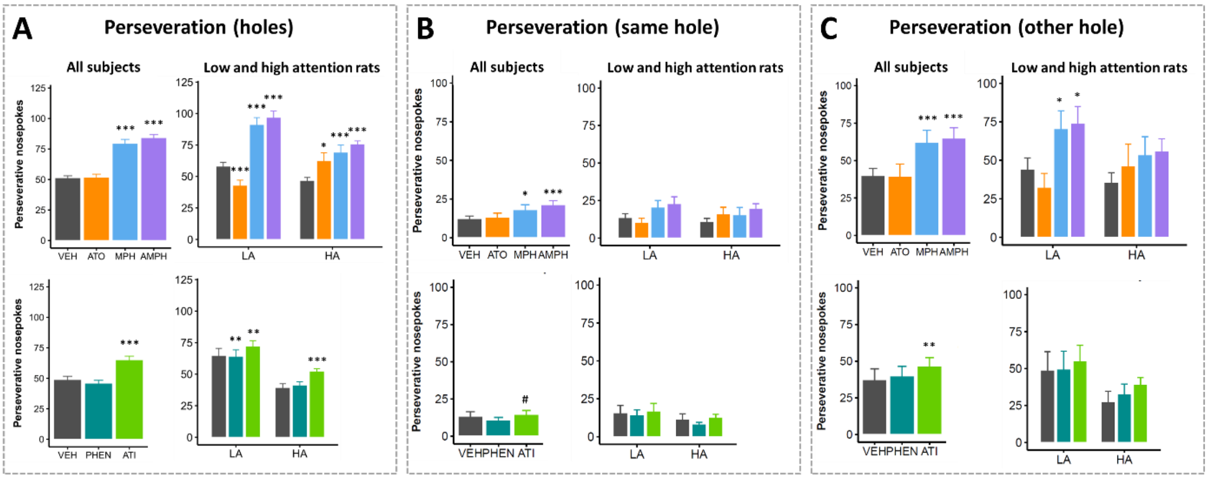

Figure S3. Standard 5CSRTT parameters perseverative nosepokes. (A-C) presents results for Latin-square 1 (LS1, top panels) and Latin-square 2 (LS2, bottom panels) with 5CSRTT parameters perseverative nosepokes overall **(A)**, in the same hole as previous response **(B)**, and in other holes than previous response **(C)**. LA, low attention rats; HA, high attention rats; ATO, atomoxetine; MPH, methylphenidate; AMPH, amphetamine; PHEN, phenylephrine; ATI, atimpamezole. Results are represented as mean ± SEM; ****p* < 0.001; ***p* < 0.01; **p* < 0.05; #*p* < 0.1.

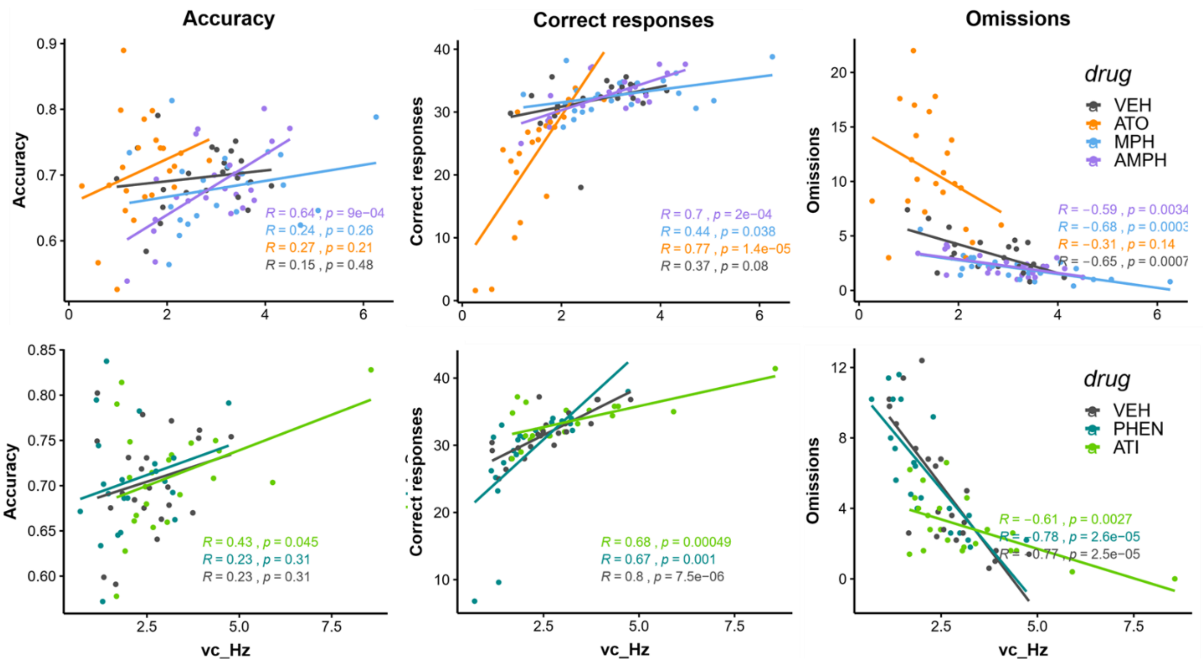

Figure S4. Correlations between ν_c and standard 5CSRTT parameters. For Latin-square 1 (top panels) and Latin-square 2 (bottom panels), we performed linear correlations (Pearson's coefficient R) TVA-modelled visual processing speed for correct responses (ν_c) and standard parameters accuracy (left), correct responses (middle), omissions (right).

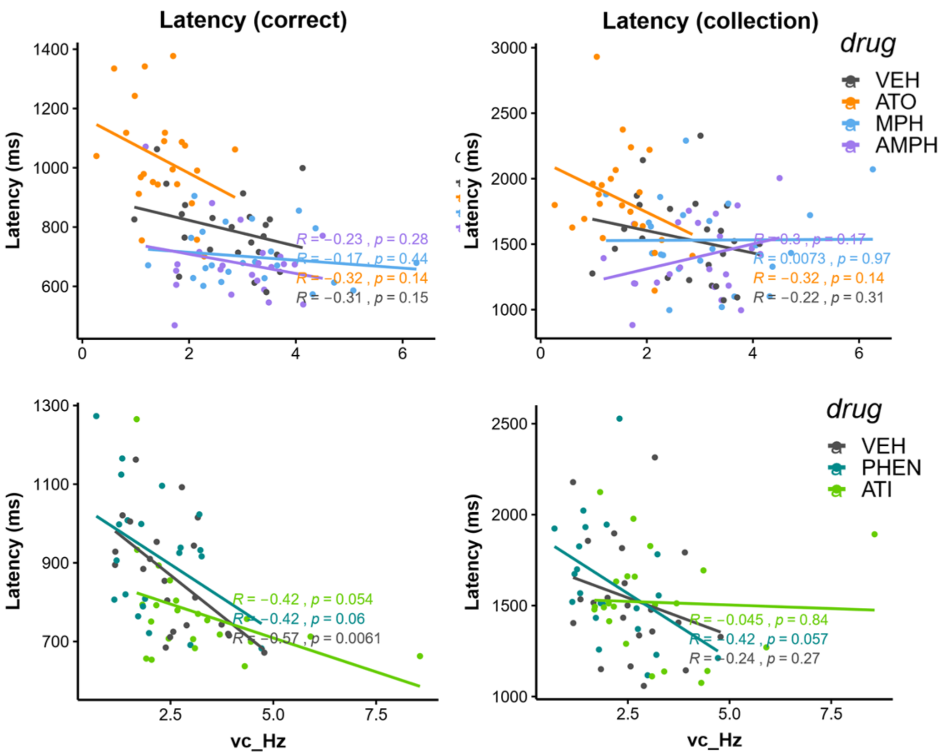

Figure S5. Correlations between ν_c and latencies. For Latin-square 1 (top panels) and Latin-square 2 (bottom panels), we performed linear correlations (Pearson's coefficient R) TVA-modelled visual processing speed for correct responses (ν_c) and standard parameters correct latency (left), reward collection latency (right).

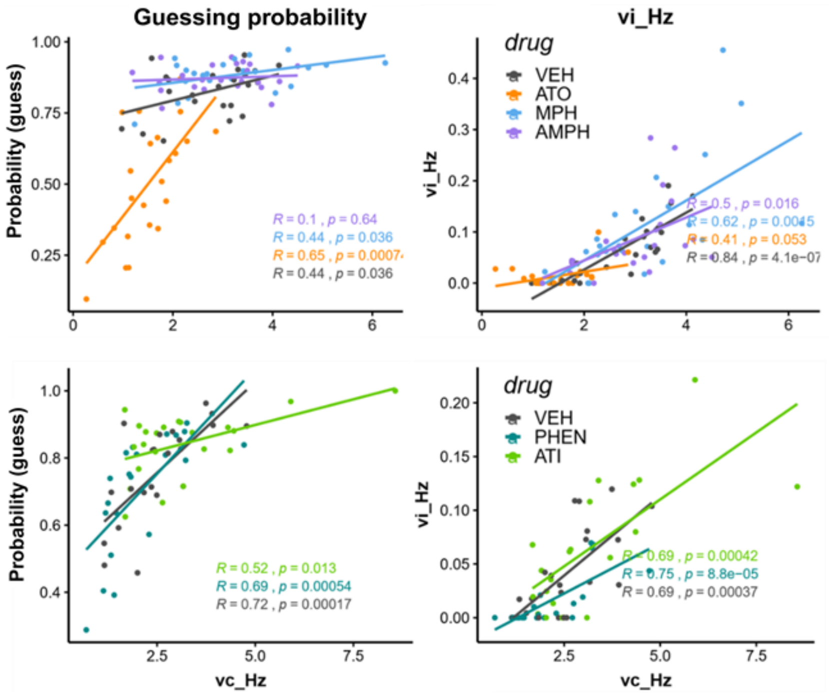

Figure S6. Correlations between ν_c and other TVA-5CSRTT parameters. For Latin-square 1 (top panels) and Latin-square 2 (bottom panels), we performed linear correlations (Pearson's coefficient R) TVA-modelled visual processing speed for correct responses (ν_c) and standard parameters correct latency (ν_i, left) and guessing probability (p_g ,right).

Table S1. Vc and Vi correlation summary

Mean VEH	v_c	v_i
Accuracy	—	—
Correct responses	↑**	—
Incorrect responses	—	—
Omissions	↓***	↓**
Premature responses	—	—
Latency correct	(↓)	—
Latency incorrect	—	—
Latency collect	—	(↓)
Perseverative NP	—	↑*
ρ_g	↑**	—
v_i	↑***	↑*

	Latin-square 1			Latin-square 2	
v_c	ATO	MPH	AMPH	PHEN	ATI
Accuracy	—	—	↑***	—	↑*
Correct responses	↑***	↑*	↑***	↑***	↑***
Incorrect responses	↑*	—	↓**	—	—
Omissions	—	↓***	↓**	↓***	↓***
Premature responses	—	—	—	—	—
Latency correct	—	—	—	(↓)	(↓)
Latency incorrect	—	—	—	—	↓*
Latency collect	—	—	—	(↓)	—
Perseverative NP	—	—	—	(↓)	—
ρ_g	↑***	↑*	—	↑***	↑*
v_i	(↑)	↑**	↑*	↑***	↑***

	Latin-square 1			Latin-square 2	
v_i	ATO	MPH	AMPH	PHEN	ATI
Accuracy	—	—	—	—	—
Correct responses	—	—	—	—	—
Incorrect responses	—	—	—	—	—
Omissions	—	(↓)	—	↓**	↓**
Premature responses	↑***	—	(↑)	—	—
Latency correct	—	—	—	—	—
Latency incorrect	↓*	—	(↓)	—	(↓)
Latency collect	—	—	—	—	—
Perseverative NP	—	—	—	—	—
ρ_g	—	—	—	↑*	↑*
v_i	(↑)	↑**	↑*	↑***	↑***

ν_c,visual processing speed for correct responses; ν_i, visual processing speed for incorrect responses; ρ_g, probability of guessing
